# COVID-19 in a Severely Immunosuppressed Patient With Life-Threatening Eosinophilic Granulomatosis With Polyangiitis

**DOI:** 10.3389/fimmu.2020.02086

**Published:** 2020-08-28

**Authors:** Markus A. Schramm, Nils Venhoff, Dirk Wagner, Jens Thiel, Daniela Huzly, Nils Craig-Mueller, Marcus Panning, Hartmut Hengel, Winfried V. Kern, Reinhard E. Voll

**Affiliations:** ^1^Department of Rheumatology and Clinical Immunology, Medical Center – University of Freiburg, Faculty of Medicine, University of Freiburg, Freiburg, Germany; ^2^Division of Infectious Diseases, Department of Internal Medicine II, Medical Center – University of Freiburg, Faculty of Medicine, University of Freiburg, Freiburg, Germany; ^3^Institute of Virology, Medical Center – University of Freiburg, Faculty of Medicine, University of Freiburg, Freiburg, Germany

**Keywords:** COVID-19, SARS-CoV-2, vasculitis, eosinophilic granulomatosis with polyangiitis, EGPA, immunosuppression, rituximab, cyclophosphamide

## Abstract

Immunosuppressive therapies increase the susceptibility of patients to infections. The current pandemic with severe acute respiratory syndrome coronavirus 2 (SARS-CoV-2) compels clinicians to develop recommendations for successful clinical management and surveillance of immunocompromised patients at high risk for severe disease progression. With only few case studies published on SARS-CoV-2 infection in patients with rheumatic diseases, we report a 25-year-old male who developed moderate coronavirus disease 2019 (COVID-19) with fever, mild dyspnea, and no major complications despite having received high-dose prednisolone, cyclophosphamide, and rituximab for the treatment of highly active, life-threatening eosinophilic granulomatosis with polyangiitis (EGPA).

## Introduction

With a wide range of clinical outcomes in coronavirus disease 2019 (COVID-19), from being asymptomatic to fatal acute respiratory distress syndrome, questions have been raised about the safety of immunosuppressive therapies (1). Individuals with anti-neutrophil cytoplasm autoantibody (ANCA)-associated vasculitides require particular care, especially considering the life-threatening course of disease with multi-organ manifestations. Pulmonary disease manifestations and immunosuppression with glucocorticoids combined with cyclophosphamide and/or rituximab are associated with infectious complications. Thus, inadequate immune response to severe acute respiratory syndrome coronavirus 2 (SARS-CoV-2) in such patients may predispose to severe COVID-19.

## Case Presentation

**FIGURE 1:**
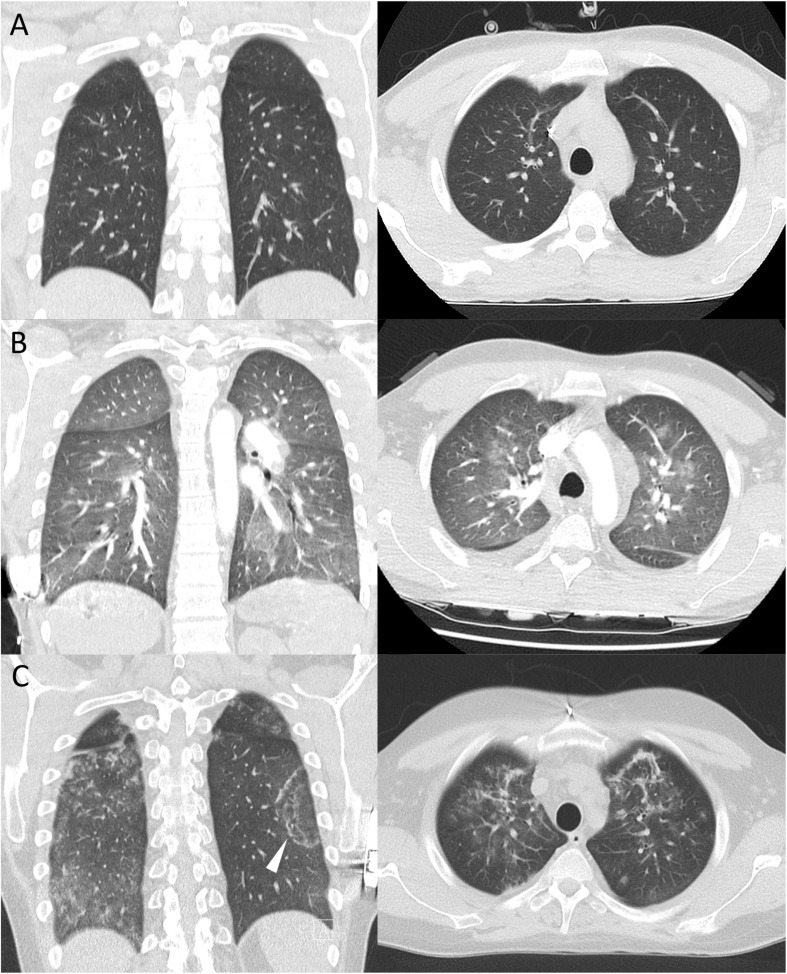
CT scans (coronal, axial) of the chest **(A)** after diagnosis of EGPA, unremarkable for pulmonary disease manifestation, **(B)** showing ground glass opacities and interlobular septal thickening after diagnosis of alveolar hemorrhage by bronchoalveolar lavage, **(C)** demonstrating bipulmonary ground glass opacities and consolidations with minor reticulation. Presence of reversed halo sign (arrow) as previously described in COVID-19 pneumonia.

We report the case of a 25-year-old male with nosocomial COVID-19 while receiving immunosuppressive treatment for eosinophilic granulomatosis with polyangiitis (EGPA). EGPA was newly diagnosed in early January 2020 when the patient presented at the emergency room with sinusitis, asthma, and a life-threatening myocardial infarction, resulting in a decreased ejection fraction of 30%. Blood eosinophils and serum concentrations of Immunoglobulin E (IgE) and C-reactive protein (CRP) were increased, ANCA-testing was negative, and a pulmonary CT scan unremarkable (Figure 1A). Immediately initiated immunosuppression with intravenous high-dose prednisolone and cyclophosphamide showed adequate therapeutic response. With conversion to oral glucocorticoid treatment at the end of January 2020, the patient unexpectedly developed a serious relapse of disease with peripheral neuropathy, pulmonary hemorrhage (Figure 1B) and a second myocardial infarction. Thus, due to severity and refractory disease the previously healthy patient was continuously hospitalized from January to March 2020, receiving intravenous cyclophosphamide (CYCLOPS-protocol, cumulative dose 4.76 g), rituximab (4 × 375 mg/m^2^), and a long-term, slowly tapered high-dose prednisolone treatment (up to 1 g/day).

**FIGURE 2:**
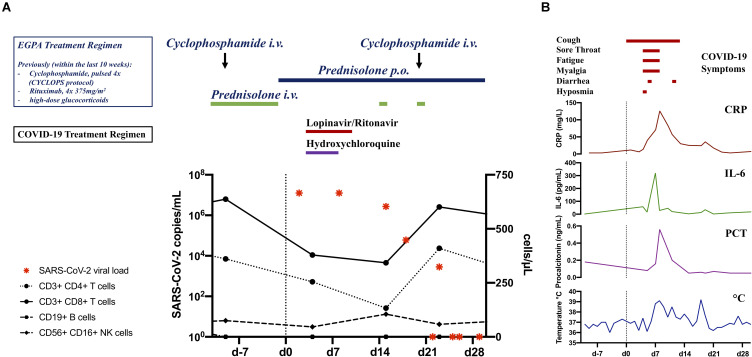
**(A)** Timeline of SARS-CoV-2 real-time reverse transcription PCR results (copy numbers) and numbers of immune cells during COVID-19. Colored bars indicate treatment regimen employed pre and post SARS-CoV-2 infection. **(B)** Course of COVID-19 induced inflammation markers quantified in patient sera including CRP, IL-6, procalcitonin (PCT), and body temperature (°C). Colored lines indicate patient symptoms related to SARS-CoV-2 infection. Vertical dashed line indicates onset of COVID-19 related symptoms.

On presumed day 0 of COVID-19 (ongoing oral treatment with 60 mg prednisolone only, 9 days after last of five cyclophosphamide infusions and 19 days after the last of four rituximab infusions), he reported catarrh and a mild cough. A SARS-CoV-2 real-time reverse transcription PCR (rt-PCR) from oropharyngeal swab was positive (Figure 2A). On day 3, treatment with hydroxychloroquine (for 6 days) and lopinavir/ritonavir (for 8 days) was initiated while daily prednisolone was reduced from 60 to 15 mg. He developed a sore throat, hyposmia, headaches, myalgias, and diarrhea. Despite rhonchi/crackles on auscultation and a CT scan consistent with bilateral viral pneumonia (Figure 1C), the patient only reported mild dyspnea. Short-term decrease of oxygen saturation (minimal SaO_2_ 85%) required oxygen supplementation for 3 days (low flow 2 L/min). With spiking serum concentrations of CRP (125.9 mg/L, reference range <5 mg/L), procalcitonin (0.56 ng/mL, reference range <0.05 ng/mL), and interleukin-6 (IL-6, 320 pg/mL, reference range <7 pg/mL), concomitant with decreasing CD4^+^ and CD8^+^ T-cell counts, the patient developed fever (max. 39.1°C) on day 7 (Figure 2B). Anti-IL6-receptor treatment was considered, however the patient steadily recovered and was free of COVID-19 symptoms 2 weeks after onset. Nevertheless, subsequent oropharyngeal swabs confirmed active SARS-CoV-2 infection with gradual decrease of viral RNA. Relapsing neurological symptoms of EGPA urged us to re-administer high-dose glucocorticoids and cyclophosphamide on days 14 and 20, respectively, without causing recurrence of COVID-19-related symptoms. Despite severe immunosuppression and complete peripheral B-cell depletion, SARS-CoV-2 RNA copy numbers in oropharyngeal swabs were below the threshold for reliable detection on days 25, 26, and 29. By day 46, there were no antibodies to SARS-CoV-2 spike protein detectable by ELISA (EUROIMMUN). Remarkably, interferon-gamma release upon polyclonal T-cell stimulation was normal on day 36.

## Discussion

Given the high-risk profile with sustained cardiac dysfunction, previous pulmonary hemorrhage, continued high-dose glucocorticoids, B-cell depletion, decreased T-cell counts, and secondary hypogammaglobulinemia (minimal IgG 4.47 g/L, reference range >7 g/L), it is remarkable that our patient overcame COVID-19 in a rather timely manner without complications. The effects of potential anti-viral agents hydroxychloroquine and lopinavir/ritonavir on the disease course remain unclear. Despite initially higher than average copies per oropharyngeal swab, which could be explained by the effect of immunosuppression during virus contraction, our patient showed a temporal pattern of viral load peaking within the first week after onset of symptoms and gradually declining over the following three weeks as previously described in COVID-19 patient cohorts (2, 3). Our observations might therefore suggest that a functional adaptive immune system with an effective B-cell response is not required to survive COVID-19. Moreover, our data points to an important role of innate immune mechanisms and perhaps T cells for SARS-CoV-2 control based on the coinciding increase of CD8^+^ and CD4^+^ T-cell numbers with declining viral RNA load. Notably, antibodies may often be insufficient for viral clearance (4). There is even evidence that antibodies against the SARS-CoV-2 spike protein can exacerbate pulmonary inflammation due to immunocomplex-mediated complement and Fcγ receptor activation with consecutive immune cell infiltration (5, 6).

While our knowledge of COVID-19 pathogenesis continues to evolve, strategies to avoid unfavorable outcomes of SARS-CoV-2 infection should continue to be mindful of potentially greater adverse outcome caused by tempering existing immunosuppressive or immunomodulatory treatment in autoimmune diseases.

## Data Availability Statement

The original data generated and analyzed for this study are included in the published article. Further inquiries can be directed to the corresponding author.

## Ethics Statement

Written informed consent was obtained from the individual for the publication of any potentially identifiable images or data included in this article.

## Author Contributions

MS and RV wrote the manuscript. All authors were involved in direct patient care or acquisition of clinical data, made important intellectual contributions, revised and approved the final version of the manuscript.

## Conflict of Interest

The authors declare that the research was conducted in the absence of any commercial or financial relationships that could be construed as a potential conflict of interest.
